# Cobalt-doped double-layer α-Fe_2_O_3_ nanorod arrays for enhanced photoelectrochemical reduction of Cr(VI)

**DOI:** 10.1186/s11671-023-03785-w

**Published:** 2023-02-10

**Authors:** Long Bai, Jueyu Wang, Kuo Yang, Yi Yan, Meitong Jin, Daizong Cui, Min Zhao

**Affiliations:** College of Life Science, Northeast Forestry University, Harbin, China

**Keywords:** α-Fe_2_O_3_ nanorods, Selective doping, Photoanode, Cr(VI) reduction

## Abstract

Element doping is an important method for improving the performance levels of photoelectrochemical (PEC) cells. Nevertheless, to date, the PEC conversion efficiency and photocurrent characteristics of the available photoanodes remain very low. In this study, cobalt (Co) was selectively doped into the bottom and/or top layers of double-layered α-Fe_2_O_3_ nanorod arrays grown on conductive transparent substrates (F:SnO_2_, FTO) via a two-step hydrothermal method; this process was performed to enhance the charge transfer ability and thus significantly improve the PEC performance. The light response capabilities of all α-Fe_2_O_3_ films were evaluated by an electrochemical workstation under dark or visible light irradiation conditions. The sample of Co doped in the bottom layer exhibited a high photoelectrochemical performance, achieving a current density of 1.37 mA/cm^2^ at + 1.0 V versus saturated calomel electrode (SCE); additionally, the sample exhibited a photoelectric synergistic ability to reduce Cr(VI) in an aqueous solution, with 84.85% reduction in 180 min. Under the influence of the electric field inside the double-layer electrode, the photoexcited electrons and holes are transferred to the surfaces of the FTO substrate and the photoanode, increasing the current density and enhancing Cr(VI) reduction. The results of this study offer an alternative approach for designing novel photoanodes with improved PEC performance levels by engineering the electron density distribution and band structure for efficient carrier separation; the results may provide new solutions in heavy metal reduction and contaminant degradation projects.

**Supplementary Information:**

The online version contains supplementary material available at 10.1186/s11671-023-03785-w.

## Introduction

Introducing a photoelectrochemical system composed of a TiO_2_ photoelectrode to split water has opened a new field for semiconductor photocatalysis research [1]. Since then, semiconductor materials, such as TiO_2_, α-Fe_2_O_3_, ZnO, WO_3_, CdS, and BiVO_4_, have been developed for use in photoelectron catalysis [2–6]. Hematite (α-Fe_2_O_3_) is one of the most promising photoelectric semiconductors because of its suitable bandgap (2.0–2.2 eV), stable phases, and abundance in nature. However, the applicability of hematite has been limited by its intrinsic properties, such as its low conductivity, short exited-state lifetime (< 10 ps), and short hole-diffusion length (2–4 nm) [7–9].

The usual approaches for overcoming these limitations and increasing the photoelectrochemical efficiency of α-Fe_2_O_3_ involve forming heterojunctions by combining two semiconductors, growing nanoparticles, and doping with foreign atoms [10, 11]. Doped elements greatly improve the conductivity of α-Fe_2_O_3_ by adding mobile charge carriers; the common doping transition metal elements include Ti^4+^, Si^4+^, Sn^4+^, Ni^2+^, and Co^2+^ [12–18]. Their use provides the most dispersed conduction band (CB) density of states, reduces the effective mass of electrons, and increases electron transfer. In addition, the optical and electrical properties of metal oxides can be improved by doping; doping involves adding external dopants to produce inherent defects. A typical example is that the photoelectrochemical (PEC) properties of Si-doped α-Fe_2_O_3_ nanofilms have improved. The optical current density exceeds 2.7 mA/cm^2^ at + 1.23 V versus reversible hydrogen electrode (RHE) [19]. Since Co and Ni are in the same group and cycle as Fe, and because the three elements have similar chemical properties, doping Co and Ni has been widely investigated; this process may increase the carrier lifetime and extend the absorption range of α-Fe_2_O_3_ photocatalysts. The in situ doping in α-Fe_2_O_3_ has demonstrated that the addition of Co or Ni minimally impacts the structure of α-Fe_2_O_3_ [20, 21]. A study of the influences of different doping elements, including Ni^2+^, Co^2+^, Ru^3+^, Ce^3+^, and Pd^2+^, on electrode performance shows that the doping of Ni and Co enhances the electrocatalytic activity of the electrode; in particular, the doping of Ni greatly improves the degradation efficiency of the electrode. This same study has confirmed that Ni and Co enter the lattice of α-Fe_2_O_3_ and increase its carrier density [22, 23]. Different doping concentrations have been shown to affect the performance of the electrode; although the carrier density increases with increasing doping content, the surrounding carrier density and the conductivity both decrease [18, 24].

The tailoring of the nanostructure of the α-Fe_2_O_3_ crystal is another important factor affecting the electrochemical characteristics and catalytic activity of the electrode; one suitable approach involves building multispacer nano-α-Fe_2_O_3_ films with large specific surface areas and good structural stabilities [25, 26]. One-dimensional α-Fe_2_O_3_ nanostructures, such as nanowires, nanorods, and nanotubes, have better optical properties and electrocatalytic performance levels because their shorter diffusion lengths help to effectively transport holes to the surface [27]. Increasing the number of array layers increases the specific surface area of the electrode and the number of catalytic active sites, tailoring the optical and electronic properties of the semiconductor. For example, a new Fe-doped NiCoP hyperbranched array structure grown on foam nickel (the secondary one-dimensional nanowire array grows on the primary two-dimensional nanosheet array) has been established; this structure maximizes the utilization of catalytic active sites and provides a large electrolyte contact surface area, significantly improving the catalytic activity [28]. Therefore, the electrocatalytic performance levels of electrode materials can be improved by using electrode materials with different structures.

By considering the above research, in this paper, a new, simple, and efficient Co-doped bilayer is prepared by hydrothermal synthesis regarding structural and doping modifications of α-Fe_2_O_3_ nanorod arrays. We construct a monolayer on the surface of fluoride-doped tin dioxide conductive glass (FTO) by a hydrothermal method. The α-Fe_2_O_3_ nanorod array structure, based on a single layer, continues to be prepared by hydrothermal synthesis technology to obtain the α-Fe_2_O_3_ electrode. This double-layer structure greatly improves the electron transfer efficiency of α-Fe_2_O_3_. Additionally, with doping modification technology as a guide, doped Co nanoparticles are used to improve the catalytic performance of the double-layer α-Fe_2_O_3_ electrode. The double layer obtained by the combination of structural and doping modifications of the α-Fe_2_O_3_ electrode has good catalytic ability and lower charge transfer impedance. This layer is applied for reducing Cr(VI) through photoelectric synergy; the reduction rate is fast and the effect is good, providing a new idea for the research and development of electrode modification.

## Experimental

### Synthesis of the α-Fe_2_O_3_ nanorod arrays

**Fig. 1:**
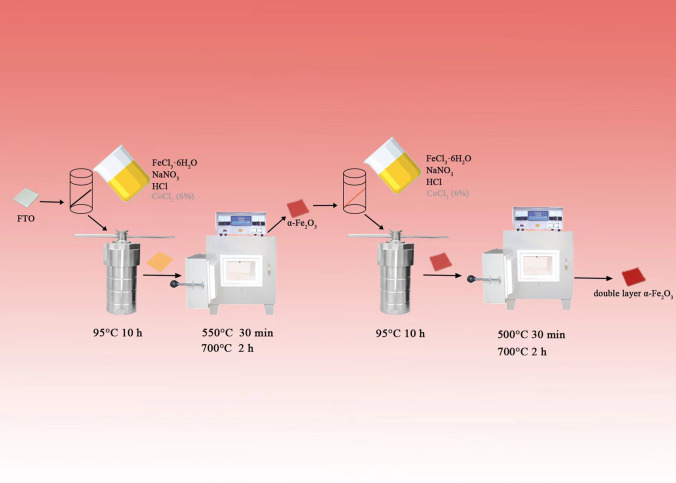
Schematic preparation of α-Fe_2_O_3_ nanorod arrays

Figure 1 shows the process flowchart for the hydrothermal synthesis of α-Fe_2_O_3_ nanorod arrays. The material was grown on fluorine-doped tin oxide (FTO; 7 Ω/sq, Top Vendor Science & Technology Co., Ltd.)-coated glass substrates using a hydrothermal method developed by Bai [29]. After being ultrasonically cleaned in deionized water and then an acetone and ethanol solution, the FTO substrate was dried with N_2_ before use; the treated FTO was stored under ethanol. To make the undoped base α-Fe_2_O_3_, 4.8653 g ferric chloride (FeCl_3_·6H_2_O, ≥ 99.0%, Tianjin Yongda Chemical Reagent Co., Ltd.) and 10.1988 g sodium nitrate (NaNO_3_, ≥ 99.0%, Tianjin Yongda Chemical Reagent Co., Ltd.) were added to 120 mL of deionized water. The pH of the mixture was adjusted to 1.5 using hydrochloric acid (HCl, 36–38 wt.%, Tianjin Yongda Chemical Reagent Co., Ltd.) and stirred for 30 min. A piece of cleaned FTO substrate was placed inside a 250 mL Teflon-lined stainless steel autoclave with its conductive side facing down; then, the solution was transferred into the autoclave and heated at 95 °C for 10 h. After cooling to room temperature, the FTO was removed from the autoclave, washed with plenty of deionized water, and dried at 70 °C for 15 min. Finally, all of the organic materials in the FTO were removed by calcination at 550 °C for 30 min under an air atmosphere in a muffle furnace (SX2, Shanghai Zhetai Machinery Manufacturing Co., Ltd.); then, the specimen was annealed at 700 °C for 2 h to form calcined α-Fe_2_O_3_ [30]. The undoped, base α-Fe_2_O_3_ nanorod arrays were labeled FTO/α-Fe_2_O_3_ (F), and the Co-doped sample was labeled FTO/α-Fe_2_O_3_:Co (FC).

### Synthesis of double-layered α-Fe_2_O_3_ photoanodes with selective Co doping

Hydrothermal processing and two-step annealing were used to prepare the Co-doped α-Fe_2_O_3_ nanorod arrays on the FTO substrate [31]; each step was almost the same as those described above. All the α-Fe_2_O_3_ nanorod arrays were doped with 6% Co (CoCl_2_, ≥ 99.7%, Sinopharm Chemical Reagent Co., Ltd.) according to the method in the literature [32]. The double-layer α-Fe_2_O_3_ nanorod arrays prepared in this manner were labeled as FTO/α-Fe_2_O_3_/α-Fe_2_O_3_ (FF), FTO/α-Fe_2_O_3_/α-Fe_2_O_3_:Co (FFC), FTO/α-Fe_2_O_3_:Co/α-Fe_2_O_3_ (FCF) and FTO/α-Fe_2_O_3_:Co/α-Fe_2_O_3_:Co (FCFC) according to the arrangement of layers.

### Material characterization

We used scanning electron microscopy (SEM; JSM-7500F) to examine the morphologies of all samples and characterized their crystal structures using powder X-ray diffraction (XRD; Bruker AXS D8 Advance diffractometer with a Cu Kα source, *λ* = 1.5406 Å). The chemical compositions of the materials were determined using X-ray photoelectron spectroscopy (XPS; Kratos Axis Ultra DLD) with monochromatic Al Kα radiation, and the charge calibration compensated for the charge effect by correcting the C 1s line of the variable carbon setting to 284.8 eV. A diffuse reflectance spectrometer (DRS; EV300 UV‒Vis, Thermo Scientific Instruments LLC, USA) was used to test the absorption spectrum of the mineral electrode, with BaSO_4_ serving as a blank for comparison.

### PEC and electrochemical characterization

Electrochemical tests were performed using a three-electrode electrochemical system on an electrochemical workstation (CHI760E, Shanghai Chenhua Instrument Co., Ltd, China) with an aqueous electrolyte solution of 0.5 M Na_2_SO_4_ at a pH of 3.0. The working electrode was the prepared hematite electrodes, a platinum foil electrode was used as the counter electrode, and a saturated calomel electrode was used as the reference electrode. Acetic acid was used as a hole-trapping agent, and the light source for simulating visible light was a 350 W Xe lamp. The optical density at the electrode surface was measured to be approximately 100 mW/cm^2^, which was sufficient to allow us to study the electrochemical properties of the bilayer nanostructures. Linear sweep voltammetry (LSV), electrochemical impedance spectroscopy (EIS), and Mott–Schottky (M–S) were used to describe the electrochemical properties of the photoanode. The starting and ending voltages of LSV were 0 V and + 1.0 V versus SCE, respectively, with a scanning speed of 0.1 mV/s and a sampling interval of 1 mV. To further understand the effect of Co doping on the charge transfer efficiency, we calculated the applied bias photon-to-current efficiency (ABPE) from the J–V curves using the following equation:

1 $${\mathrm{ABPE}}={I}\times (1.23 - |V|)/P$$

where* I* is the photocurrent density at potential *V* and *P* is the simulated light intensity (100 mW/cm^2^).

The EIS potential was established as the open circuit voltage (OCP) of the working electrode, the frequency range was 10^5^ Hz to 1 Hz, and a 5 mV amplitude. The EIS test data of the anode under different experimental conditions were sorted as the Bode diagram, while the polarization internal resistance (RP) of the system was calculated using the equivalent circuit simulation program built in the instrument. M–S measurements were performed in a three-electrode configuration at a frequency of 1 kHz, and the start and end potentials were set in the range of 0.5–1.0 V for the open circuit voltage and 0.01 V for the amplitude; the slope from the M–S plots was used to estimate the carrier densities (Nd) in semiconductors using the following equation:

2 $$1/C_{\text{sc}}^{2}= \left[2/e{\varepsilon}_{r}{\varepsilon }_{0} {N}_{d}{A}^{2}\right]\left(E-{E}_{\text{fb}}- kT/e\right)$$

where *C*_sc_ is the capacitance, *e* is the electron charge (1.602 × 10^−19^ C), *ε*_*r*_ is the vacuum permittivity (8.854 × 10^−12^ F/cm), *ε*_0_ is the dielectric constant of the material (80), *A* is the working electrode area (25 cm^2^), *E* is the electrode potential, *E*_fb_ is the flat band potential, *k* is the Boltzmann constant (1.38 × 10^−23^ J/K), and *T* is the thermodynamic temperature.

### Reduction of Cr(VI) by the photoelectric electrodes

Amperometric i–t curve (i–t) and CV were used to evaluate the effects of oxygen, the cavity trapping agent, and the pH level on chromium reduction, and N_2_ was introduced 30 min before the test to purify the dissolved oxygen system for the experimental group. I–t curves were measured at + 0.5 V versus SCE, while the pH level of the reaction system was adjusted to 3–5 with hydrochloric acid; the CV parameters were set to a potential range of − 0.8 to + 0.5 V. The scanning speed was 100 mV/s (reverse scanning), and continuous cyclic scanning was performed until the curve was stabilized. The decrease in Cr(VI) concentration with time was monitored spectrophotometrically at 540 nm according to the approach adopted by Bennett [33]. The measurements were initiated after mixing the chromium reagent 1,5-diphenylcarbohydrazide with 5 mL of the filtrate for 5 min. The reduction rate of Cr(VI) was calculated using the following formula:

3 $${R}=\left({c}_{0}-{c}_{t}\right)/ {c}_{0}\times 100\%$$

The CV curve was used to test the reduction of Cr(VI) every 20 min. In contrast, the rest of the test conditions remained the same for the electrochemical technology as previously, and the voltage range was changed to − 0.8 to 0 V versus SCE.

## Results and discussion

### Morphology

**Fig. 2:**
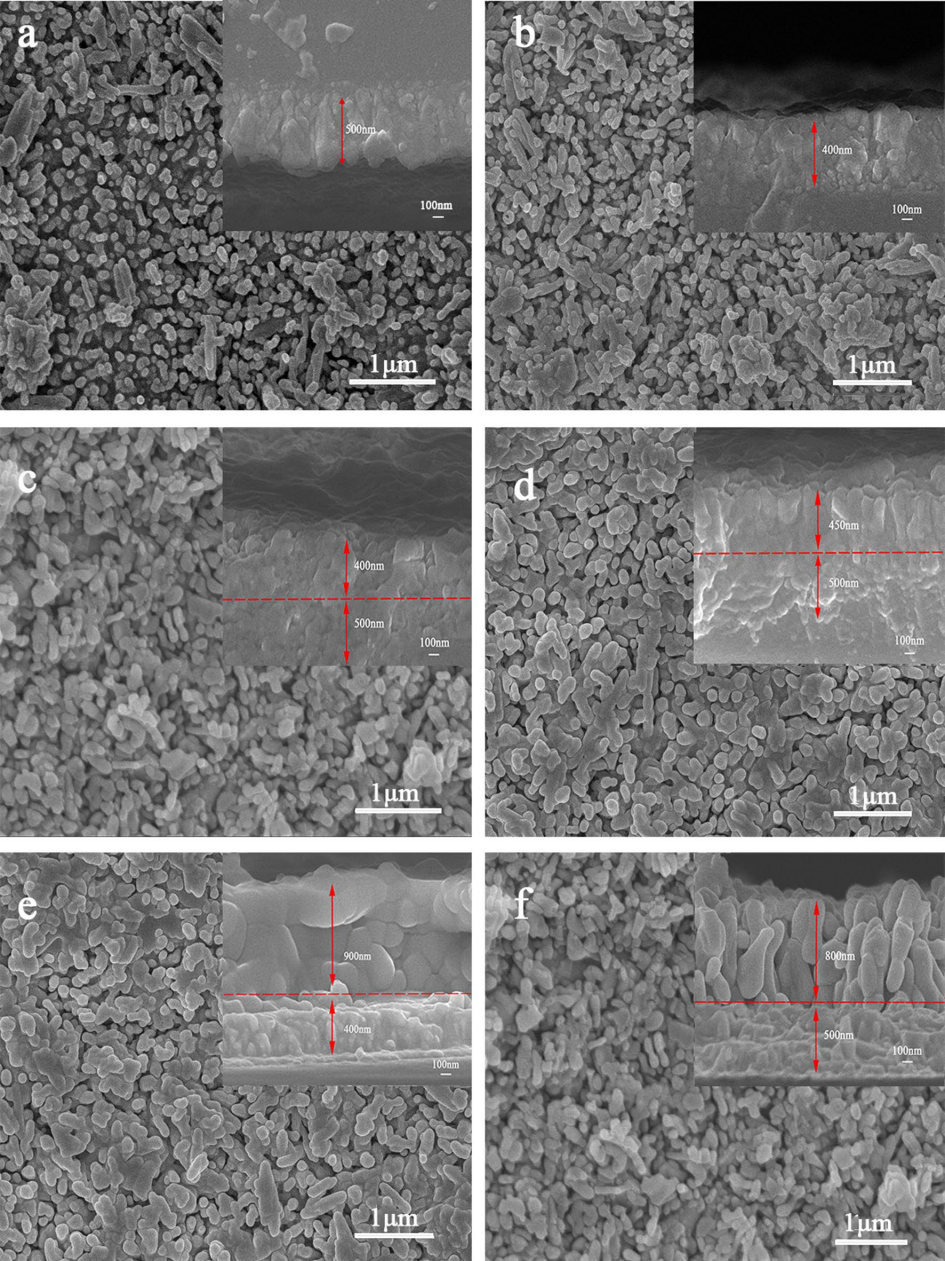
SEM of **a** FTO/α-Fe_2_O_3_, **b** FTO/α-Fe_2_O_3_:Co, **c** FTO/α-Fe_2_O_3_/α-Fe_2_O_3_, **d** FTO/α-Fe_2_O_3_:Co/α-Fe_2_O_3_, **e** FTO/α-Fe_2_O_3_/α-Fe_2_O_3_:Co, and **f** FTO/α-Fe_2_O_3_:Co/ α-Fe_2_O_3_:Co

The double-layered α-Fe_2_O_3_ nanorod arrays were fabricated via a two-step hydrothermal method, and the single-layered undoped and co-doped α-Fe_2_O_3_ nanorods using a one-step method as a reference. After annealing at 700 °C for 2 h, the α-Fe_2_O_3_ films turn red (Supplementary Fig. S1). The top- (Fig. 2) and side-view (inset of Fig. 2) SEM images show that the doped and undoped thin films comprise numerous nanorods with average lengths of 400–900 nm and diameters of approximately 50 nm. It is easily acceptable that the thickness of the bottom α-Fe_2_O_3_ layer is similar to that of the single-layered α-Fe_2_O_3_ film, given the same aqueous growth process as reported in a previous study [34]. The nanorod lengths increase with increasing doping in double-layered nanorods (Fig. 2c-f). The morphologies of the single and double layers are very similar in the nanorods from all groups, with the only difference being the thickness. The uniform distribution of vertically aligned nanorods across the FTO substrate demonstrates the suitability of the chosen synthesis approach. Representative morphology changes due to the doping elements are shown in the SEM images in Fig. 2b. With Co doping, the layer thickness decreases from 500 nm (Fig. 2a, F) to 400 nm (Fig. 2b, FC). However, the underlying films have the same crystal structure, thickness, and composition (Fe, O, and Si) as the single-layer film; thus, the probable cause is the different substrate, as far as we know that the film thickness plays a trade-off effect on optical absorption and charge carrier transport [20]. With F or FC as the substrate, the thickness of the upper film increases significantly (Fig. 2c–f). By comparing the SEM photographs and results in the literature, the film thicknesses measured from the cross section should be reliable.

In this study, the three factors that affect the semiconductor performance are the thickness of the as-synthesized film, the degree of Co doping, and whether the film is a monolayer or bilayer. The above results indicate that Co is successfully doped into the α-Fe_2_O_3_ nanorod films and changes the carrier mobility characteristics of the films. Figure 6 shows the schematic energy diagram of Co-doped double-layered α-Fe_2_O_3_ photoanodes; research shows that the increase in the charge carrier density increases the Fermi level and that the different Fermi energy levels of the bilayer films lead to band bending, improving the charge transfer ability [35]. In addition, the bilayer structure increases the specific surface area of the sample. Thus, these Co-doped α-Fe_2_O_3_ thin films show better electrochemical properties than single-layer α-Fe_2_O_3_ nanorod films.

### Characterization of the α-Fe_2_O_3_ nanorod arrays

**Fig. 3:**
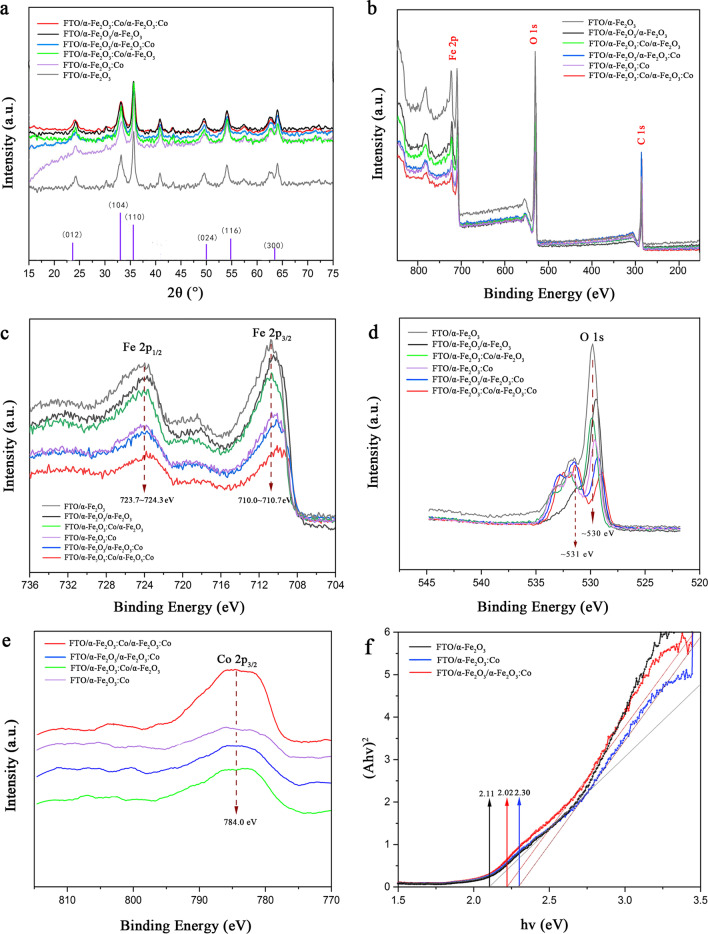
**a** XRD patterns **b** XPS survey **c** Fe 2p XPS core-level spectrum **d** Co 2p XPS core-level spectrum **e** O 1s XPS core-level spectrum, and **f** UV–Vis DRS of different photoelectrodes

XRD and XPS were used to confirm the structure and chemical states of the modified electrodes. Figure 3a shows the XRD patterns of different electrodes. According to the XRD patterns, the diffraction peaks are 24.1°, 33.1°, 35.6°, 49.4°, 54.0°, and 63.9°, and the corresponding substances are the α-Fe_2_O_3_ phase (JCPDS card No.85-0599), (012), (104), (110), (024), (116), and (300) crystal planes, respectively [36, 37]. The diffraction peaks found in the XRD patterns match those of the hematite phase, indicating pure phases of α-Fe_2_O_3_ in these double-layered photoanodes. The relatively high intensity of the (110) peak designates a strong preferential orientation of the (110) axis vertical to the substrate. The peak value of the diffraction peak of (110) crystal plane is the highest, indicating that the prepared hematite mainly exposes (110) crystal plane, and the (110) crystal plane is more conducive to the charge of the photogenerated carrier [38]. Co doping does not affect the change of the α-Fe_2_O_3_ phase. However, a careful examination demonstrates that the peak intensities associated with the (012), (104), (116), and (300) planes slightly decrease with Co doping into the α-Fe_2_O_3_ layers; this phenomenon is likely related to Co doping inhibiting the growth of the nanorods along the (110) direction, changing the film thickness, as observed in the SEM images. To confirm the existence of Fe, Co, and O elements in the samples and their oxidation state for later analysis of PEC performance and mechanism, the XPS spectrum was performed as shown in Fig. 3b–e and Fig. S2–S4. The XPS results show that obtained the main product comprises Fe and O (Fig. 3b). Figure 3c displays the Fe 2p XPS spectra of the different photoanodes; the two strong features of Fe 2p_3/2_ and Fe 2p_1/2_ are observed, which are consistent with the literature [39, 40], corresponding to Fe^3+^ in α-Fe_2_O_3_ nanorods; the lack of a Fe^2+^ satellite peak at 715.5 eV shows that there are no Fe^2+^ impurities in these samples. Although the Fe 2p_3/2_ and Fe 2p_1/2_ peaks of all the photoanodes show very similar profiles, the Fe 2p_3/2_ peak of FF is 710.8 eV, FCF 710.6 eV, FFC 710.4 eV, and FCFC 710.0 eV in Fig. S2, indicating that the doping of Co causes the peak position of Fe 2p to move forward. Figure 3d shows the O 1s spectra of the α-Fe_2_O_3_ were fitted into two peaks at approximately 530.2 eV and 531.4 eV, corresponding to lattice oxygen species of α-Fe_2_O_3_ and FTO, respectively. Moreover, Fig. 3d displays the Co 2p_3/2_ peaks of all of Co doing photoanodes the binding energy of 784.0 eV indicates the Co 2p_3/2_ peaks revealing that the oxidation state of Co is Co 2p. Co doping and the two-step method may change the peaks of O 1s and Co 2p_3/2_ slightly (Fig. S3, S4). The O 1s spectra of FF and F have two peaks, but there are three in FCF, FFC, FFFC, and FCFC, indicating that the existence of Co affects the Fe–O bond, producing Co–O bond [41, 42]. These results clearly confirm the transfer of electrons from Co to α-Fe_2_O_3_, improving the carrier transfer efficiency regarding photocatalytic performance [43, 44].

Figure 3f and Fig. S5 show the DRS spectra of several α-Fe_2_O_3_ films. These samples show good optical absorption characteristics in the UV visible region (200–800 nm), and the absorption edges of the FCF, FFC, and FC samples are significantly redshifted; this phenomenon may have occurred due to the existence of lattice defects and oxygen vacancies caused by the double-layer structure and the addition of cobalt. The intersection of the tangent of the absorption curve with the X-axis is the absorption threshold (*λ*g) of the nanoparticles. According to the transformed Kubelka–Munk function, the band gap (*E*_g_) = 1240/*λ*, and the corresponding band gaps of F, FC, FF, FCF, FFC, and FCFC are estimated to be 2.11, 2.10, 2.30, 2.02, 2.00, and 2.15 eV, respectively; thus, the decrease in the band gap observed here may be attributed to the high surface energies of the nanoparticles [45]. Based on the above experimental results, the element doping process and the bilayer structure may have a synergistic effect, improving the carrier transfer efficiency and reducing the width of the band gap of Co-doped α-Fe_2_O_3_; these aspects improve its photocatalytic performance under visible light.

### Electrochemical properties of the α-Fe_2_O_3_ films

**Fig. 4:**
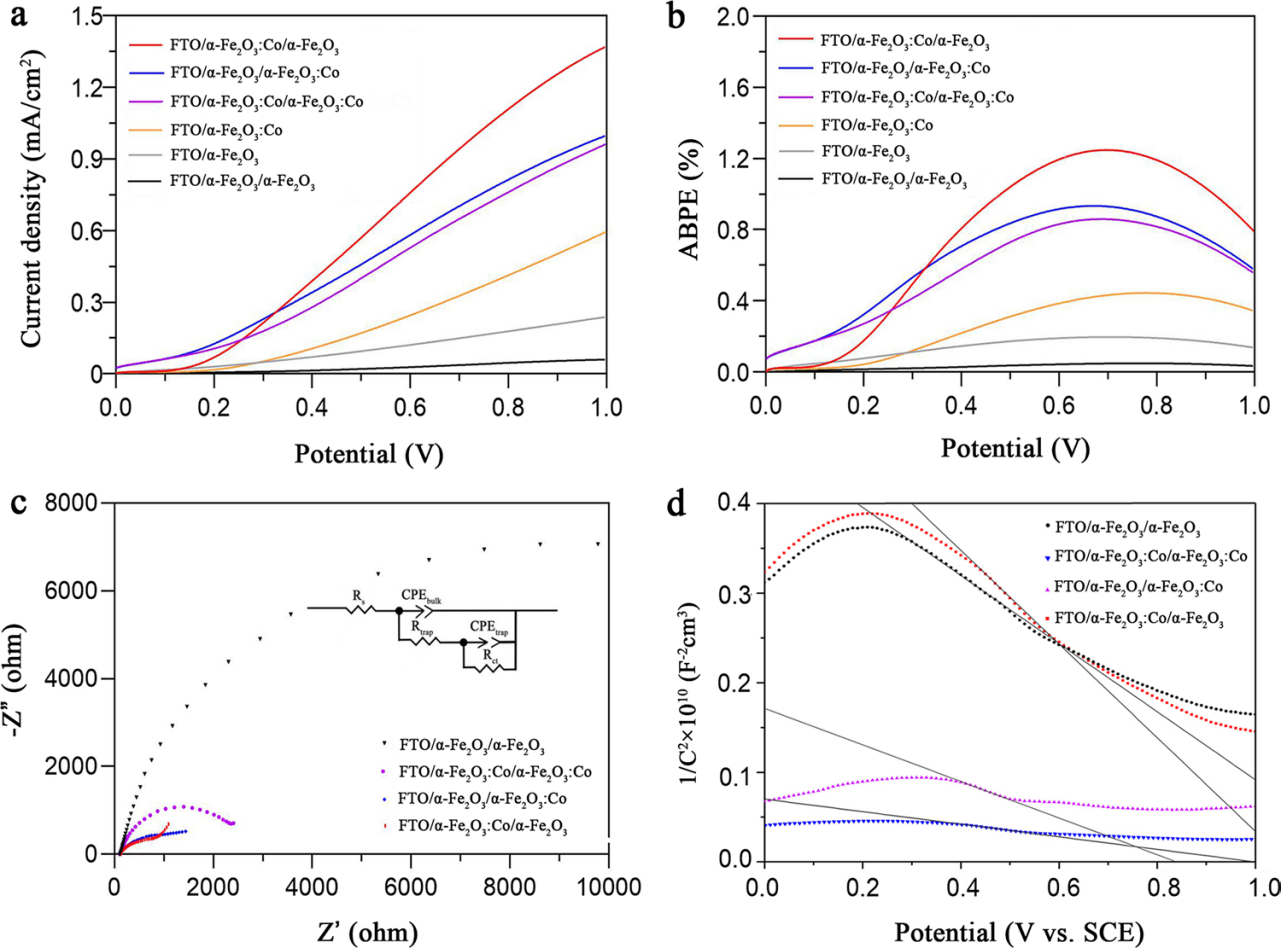
**a** LSV, **b** ABPE, **c** Nyquist plots, and **d** Mott–Schottky of different photoelectrodes

For the redox reaction of semiconductor photoelectrodes to occur, there must be a separation of electron–hole pairs on their surface [46]. The redox reactions that occur on the surfaces of semiconductor photoelectrodes usually originate from the semiconductor–solution interface and with external biasing. The electrochemical test results using α-Fe_2_O_3_ nanorod arrays as working electrodes are as follows:

LSV (I–V curves) is used to characterize the electrochemical properties of the photoanodes and to study the charge carrier characteristics at the semiconductor–electrolyte interface. Figure 4a shows the LSV collected from the undoped and doped α-Fe_2_O_3_ electrodes. For the undoped α-Fe_2_O_3_ photoanode, the current density is approximately 0.24 mA/cm^2^; the density increases slightly with element doping (the FC photoanode reaches 0.59 mA/cm^2^), while all Co-doped photoanodes show more obvious light responses. This phenomenon is attributed to the improvement in visible light absorption and suitable interfacial charge transfer on the doped α-Fe_2_O_3_. Significantly, the FCF sample shows a maximum photocurrent density of 1.37 mA/cm^2^ at + 1.0 V versus SCE; this value is an enhancement exceeding 2285%, 143%, and 137% relative to the FF (60 µA/cm^2^), FCFC (0.96 mA/cm^2^), and FFC (0.998 mA/cm^2^) samples, respectively (Table S1 in the Supplementary Materials). This current density is much higher than those seen with the single-layer electrode and doping metal element electrodes; this value has more advantages than some Fe_2_O_3_ electrodes doped with metal elements (Table S2). The decrease in current density seen with the FF electrode may occur due to the thickening of the film, hindering electron transmission; this phenomenon is attributed to the porosity of the film [47]. The photogenerated holes on the electrode surface participate in the water oxidation reaction; therefore, several studies reported in the literature have estimated the PEC water hydrolysis light conversion efficiencies (ABPE) for these photoanodes (Fig. 4b and Table S1). The α-Fe_2_O_3_ materials prepared in this study show better photoconversion efficiencies than those reported at 0.657 V, 0.775 V, and 0.687 V for the FF (0.09%), FFC (0.93%), and FCFC (0.86%) electrodes, respectively. The FCF electrode exhibits an optimal photoconversion efficiency of 1.25% (0.701 V), which is more than 1.34 times and 1.45 times greater than those of the FFC and FCF electrodes, respectively. The ABPE and current density at + 1.0 V versus SCE of the FCF electrode are higher than those of Maitra et al. [48] (Table S2); this FCF photoelectrode shows better performance regarding the light response.

Based on the above results, we perform EIS and M–S using double-layer electrodes to elucidate the effects of Co-selective doping on the electronic properties of double-layered α-Fe_2_O_3_ nanorod films. In the obtained Nyquist diagram (Fig. 4c), Z′ and Z″ are the real and imaginary parts of the impedance spectrum (z), respectively; these values illustrate the electron transfer dynamics of the FF, FFC, FCFC, and FCF electrodes. All the curves start with a half ring and then increase along the virtual impedance axis. The half-ring diameter of the FCF electrode is the smallest, indicating that the charge transfer speed of the FCF is the fastest. This phenomenon shows the equivalent circuit obtained from EIS spectrum analysis using ZSimpWin software (Ametek SI). In the equivalent circuit, the solution resistance (Rs) and charge transfer resistance (Rct) represent the internal and transfer resistance characteristics of the double-layer electrode, respectively. Rs represents the comprehensive resistance and includes the ionic resistance of the electrolyte, the inherent resistance of the electrode, and the contact resistance of the active material–collector interface. There is a Faraday reaction related to Rct. The calculated values of Rs and Rct are given in Table S3, and they show that the FCF electrode has lower Rs and Rct values than the FF, FFC, and FCFC electrodes, indicating faster reaction kinetics.

The slopes of the Mott–Schottky plots of the α-Fe_2_O_3_ electrodes (Fig. 4d) demonstrate their p-type semiconductor properties. Notably, the slopes of the remaining groups are smaller than those of α-Fe_2_O_3_; the smaller the slope is, the higher the electron density. The charge carrier density of FCF calculated using the Mott–Schottky equation is almost one order of magnitude higher than those of other electrodes (2.51 × 10^21^ cm^−3^, 3.35 × 10^20^ cm^−3^ (FF), 3.22 × 10^20^ cm^−3^ (FFC), and 8.62 × 10^20^ cm^−3^ (FCFC)) (Table S4). Clearly, the carrier densities significantly increase with the introduction of Co dopants; this phenomenon should give rise to the increased electrical conductivity and then the improved charge transfer ability [12] and is one of the reasons for PEC performance enhancement.

### Photocatalytic performance levels of the α-Fe_2_O_3_ films

The amount of dissolved oxygen and the pH level are important factors affecting the reduction efficiency in the photoelectric reduction of Cr(VI) [49, 50]. Reportedly, if there is dissolved oxygen in the reaction system, the electron transfer resistance (Rs) of the electrolyte and the surface resistance of the electrode increase significantly, hindering electron transfer. As the reaction of photogenerated electrons with dissolved oxygen in the system significantly reduces the reduction of Cr(VI), the influences of dissolved oxygen on the performance levels of the electrodes must be evaluated.

**Fig. 5:**
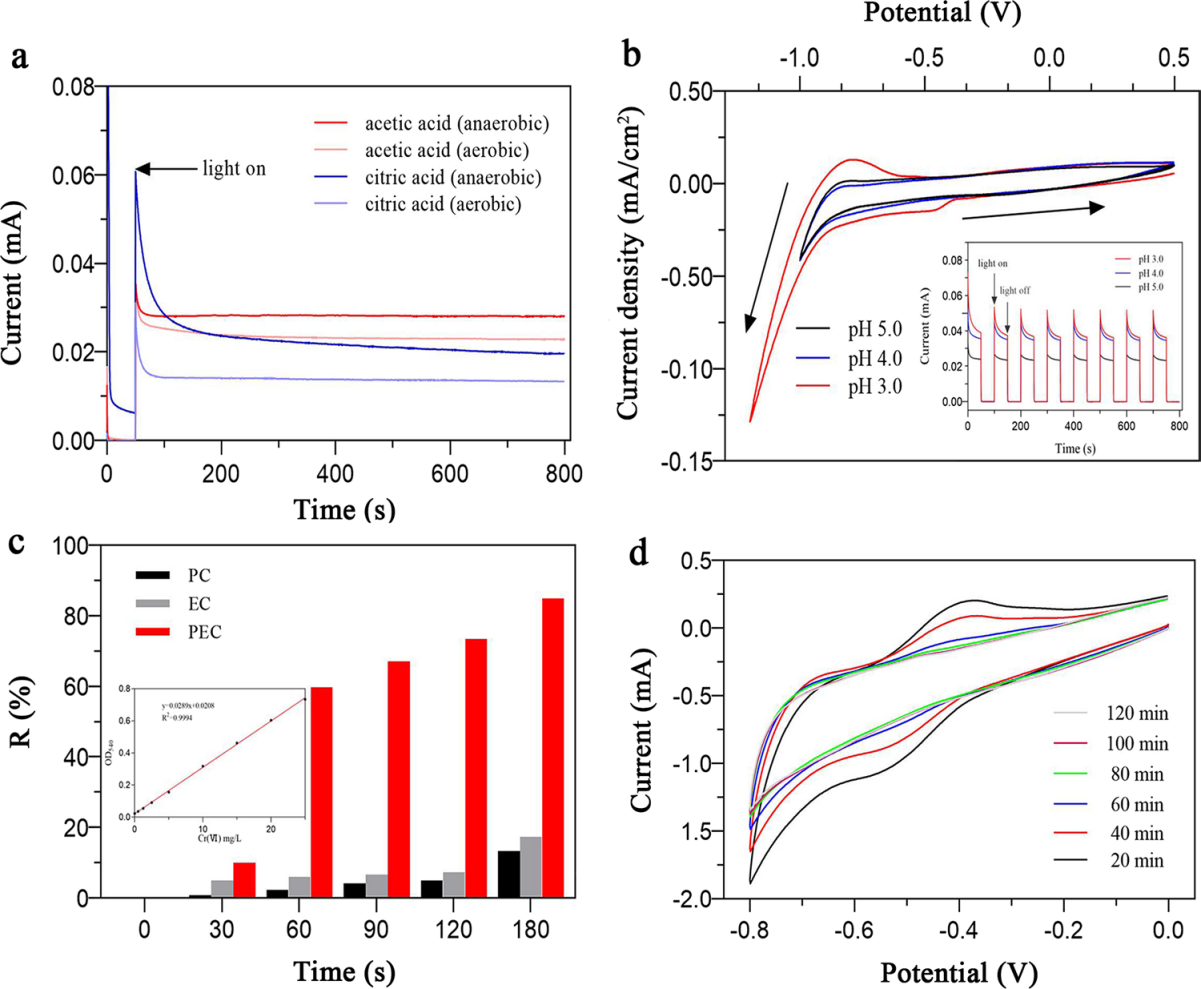
**a** Effect of oxygen and organic acid on the i–t curve, **b** CV and transient photocurrent measured at different pH, **c** reduction of chromium, and **d** cyclic voltammetry curve of chromium reduction

Since the FCF electrode shows the best electrochemical performance in our tests, we have chosen it for this part of the study. As Fig. 5a shows that after the dissolved oxygen is removed, the photocurrent of the system increases significantly; this phenomenon may accelerate the rate of reduction in Cr(VI), acetic acid has a better effect as a hole-trapping agent. As Fig. 5b shows, the rate of reduction in Cr(VI) under different pH conditions is best at a pH level of 3.0. The state of Cr(VI) is closely related to the pH of the system and the species and concentrations of other ions present. At low pH levels, there are many protons in the solution that compete with Cr(VI) for electrons, affecting the reduction of Cr(VI). The protonated and positively charged photocatalyst surface is more conducive to the adsorption of HCrO_4_^−^, greatly promoting the reduction of Cr(VI) at lower pH values. The decrease in the positive charge and the increase in the negative charge on the surface of the photocatalyst due to the increase in pH inhibit the adsorption of CrO_4_^2−^, weakening the reduction of Cr(VI). The above results indicate that the reduction of Cr(VI) is best at pH 3.0 and with a deaerated solution.

The effects of the different PEC, electrochemical (EC), and photocatalytic (PC) processes on the reduction of Cr(VI) are summarized in Fig. 5c. Under PC treatment conditions (light only), approximately 2.3% of the Cr(VI) is removed after 1 h. The application of an external bias field, *E*_bias_, increases the removal of Cr(VI) significantly, and the removal is further accelerated by simultaneous irradiation. As Fig. 5c shows, EC treatment (*E*_bias_ = + 1.0 V vs. SCE) for 1 h removes approximately 6.0% of the Cr(VI); the PEC treatment group removes up to 59.8% of the Cr(VI) for the electrode at + 1.0 V versus SCE. The reduction rates of the Cr(VI) solution that is treated for 180 min using PC, EC, and PEC are 13.3%, 17.3%, and 84.9%, respectively (Table S5). Under irradiation conditions, a small *E*_bias_ drives the effective separation of photogenerated charges, which is recombined without an *E*_bias_. Figure 5d shows the CV curve of Cr(VI) reduction by the FCF electrode over 180 min. The redox peak gradually flattens as the reaction proceeds, corresponding to the reduction of Cr(VI).

### Mechanism for the enhanced photocatalytic activity of the doped double-layer materials

**Fig. 6:**
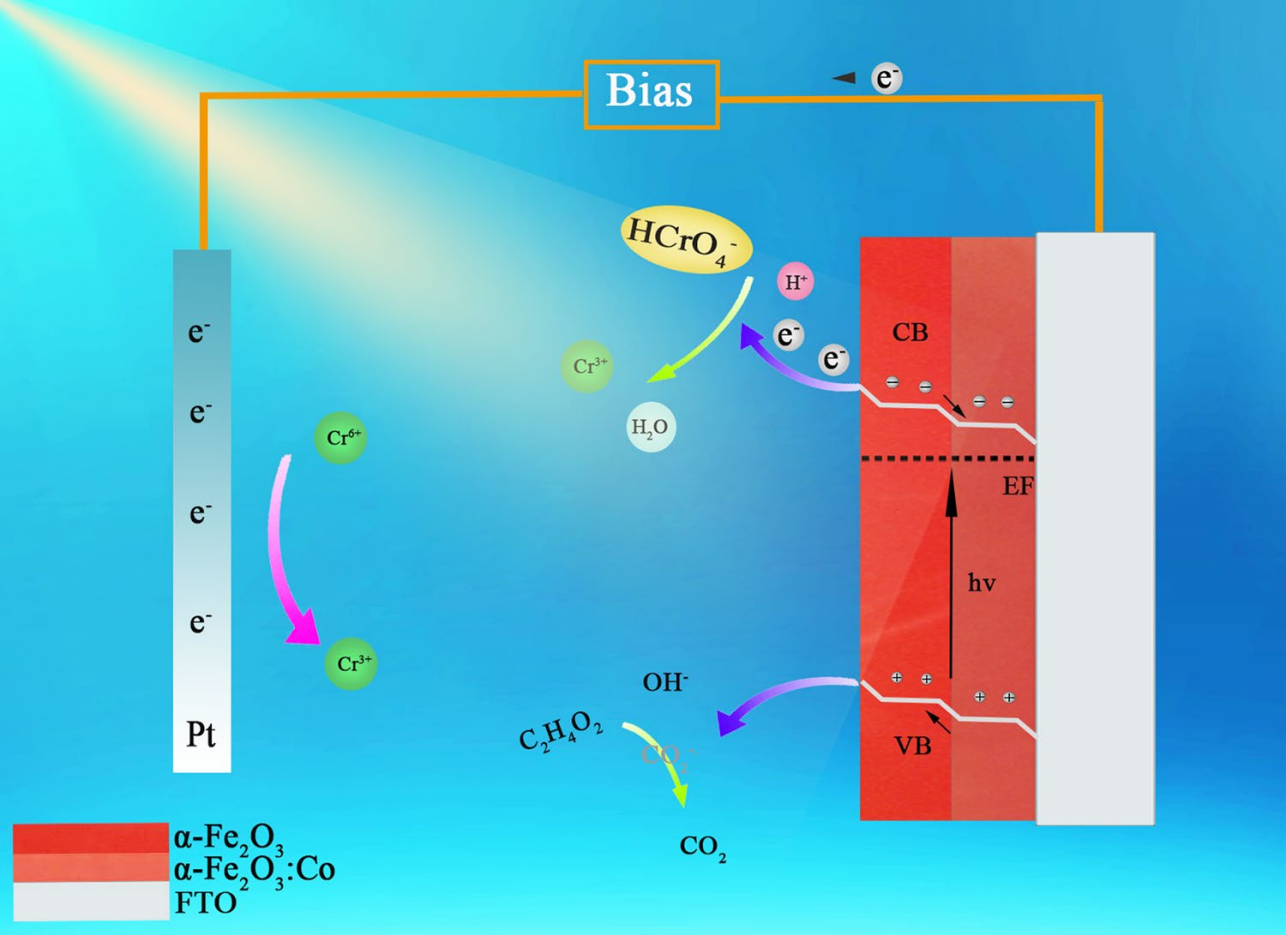
Schematic diagram of double-layer photoelectrode enhancing chromium reduction

Theory indicates that interstitial Co ions acting as electron donors increase the electron density, improving the Fermi levels of semiconductors [51]. Based on the similar valence band (VB) positions detected by XRD and UV‒Vis DRS and relevant research scholars [35], it is inferred that Co doping changes only the Fermi level of α-Fe_2_O_3_ and not the band structure. The Fermi levels of the bilayer α-Fe_2_O_3_ photoanodes in which both layers are doped with Co are higher than those of the undoped FF electrodes. However, if the bottom and top layers are both doped with Co, the increasing Fermi level does not drive the electron flow [35]. Rather, the selective doping of Co results in different Fermi levels between the two layers, and the bending of the energy band promotes the separation of photoexcited electrons and holes; these electrons and holes are further transferred to the counter electrode and photoanode surface to participate in water redox reactions, improving the PEC performance [52, 53]. This enhanced PEC performance accelerates the formation of hydroxyl radicals from hole-oxidized water, which is conducive to the reduction of Cr(VI) to Cr(III) (Fig. 6). The photoexcited electrons (e^−^) are simultaneously transferred to the cathode through an external loop, where they drive the reduction of Cr(VI).

In this study, we have demonstrated that Co doping and the growth of a double-layer nanostructure improve PEC performance by improving the photocurrent density and ABPE, enhancing the reduction of Cr(VI). Additionally, we recognize that the reduction ability achieved here is lower than that of many other photocatalytic studies. Therefore, the PEC performance of doped double-layer materials must be further improved through various approaches, such as cocatalyst decoration and heterostructure design.

## Conclusions

In summary, Co-doped α-Fe_2_O_3_ nanorod arrays have been successfully prepared through a simple and facile two-step method on a conductive transparent substrate composed of FTO. The Co-doped photoanode in the bottom layer (the FCF electrode) exhibits the highest PEC performance; when the applied voltage is + 1.0 V versus SCE, the current density reaches 1.37 mA/cm^2^ under 350 W Xe lamp illumination, which is 22.85 times that of the undoped α-Fe_2_O_3_/α-Fe_2_O_3_ nanorod film (0.06 mA/cm^2^) and even much higher than that of the α-Fe_2_O_3_ nanorod film with both Co-doped layers. Since the doping of Co increases the α-Fe_2_O_3_ electron density and the bilayer structure increases the specific surface area of the sample, the Fermi energy level in the α-Fe_2_O_3_:Co layer improves, effectively enhancing the photocurrent response. This phenomenon causes these electrodes to display an enhanced capacity for the PEC reduction of Cr(VI). In this work, we demonstrate that doping Co into the anode and establishing a double-layer structure improve the performance of the photoelectric pole and the applicability of semiconductor mineral photoelectrodes in the future.

## Supplementary Information

Supplementary file 1.

## Data Availability

All data generated or analyzed during this study are included in this published article.
