# Supplementary material for: Cobalt-doped double-layer α-Fe2O3 nanorod arrays for enhanced photoelectrochemical reduction of Cr(VI)
Source: Discov Nano. 2023 Feb 10;18(1):10. doi: 10.1186/s11671-023-03785-w (PMC9918706; doi:10.1186/s11671-023-03785-w)
Supplement: Supplementary file 1 [file 11671_2023_3785_MOESM1_ESM.pdf]

Cobalt-doped double-layer α-Fe_2O_3 nanorod arrays for enhanced photoelectrochemical reduction of Cr(VI)

Long Bai[#], Jueyu Wang[#], Kuo Yang, Yi Yan, Meitong Jin, Daizong Cui*, Min Zhao*

College of Life Science, Northeast Forestry University, Harbin, China

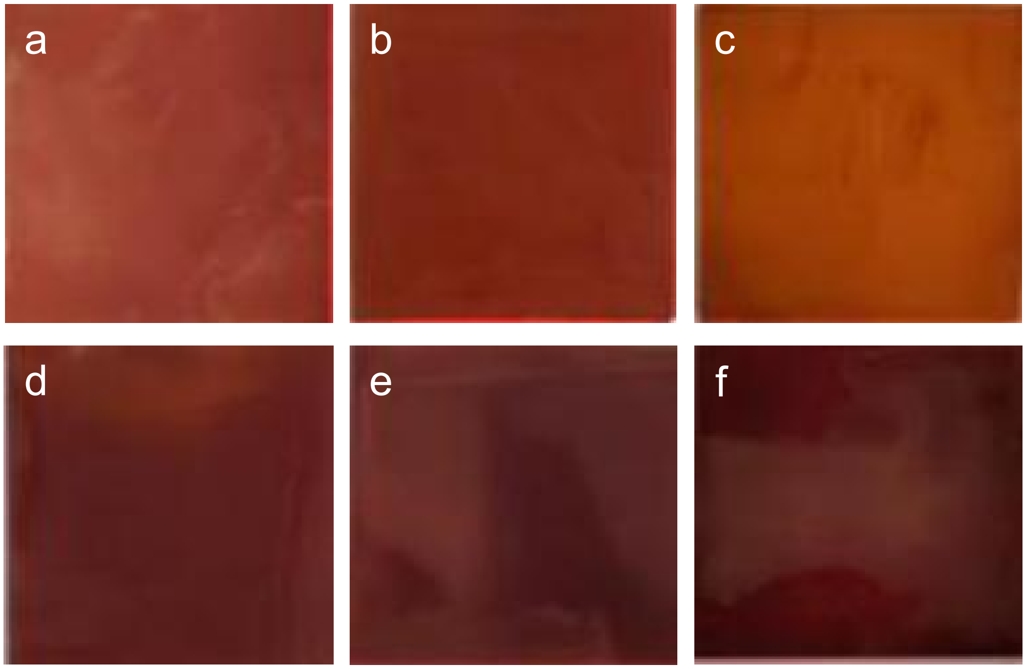

Fig. S1 Pictures of (a) FTO/α-Fe_2O_3, (b) FTO/α-Fe_2O_3:Co, (c) FTO/α-Fe_2O_3/α-Fe_2O_3, (d) FTO/α-Fe_2O_3/α-Fe_2O_3:Co, (e) FTO/α-Fe_2O_3:Co/α-Fe_2O_3, and (f) FTO/α-Fe_2O_3:Co/α-Fe_2O_3:Co

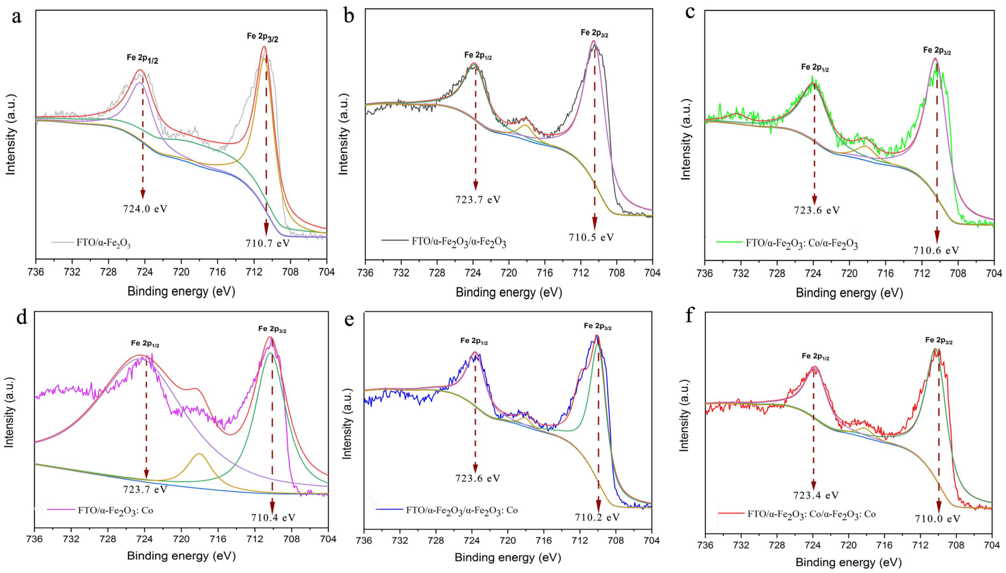

Fig. S2 Fe 2p XPS of (a) FTO/α-Fe_2O_3, (b) FTO/α-Fe_2O_3/α-Fe_2O_3, (c) FTO/α-Fe_2O_3:Co/α-Fe_2O_3, (d) FTO/α-Fe_2O_3:Co, (e) FTO/α-Fe_2O_3/α-Fe_2O_3:Co, and (f) FTO/α-Fe_2O_3:Co/α-Fe_2O_3:Co

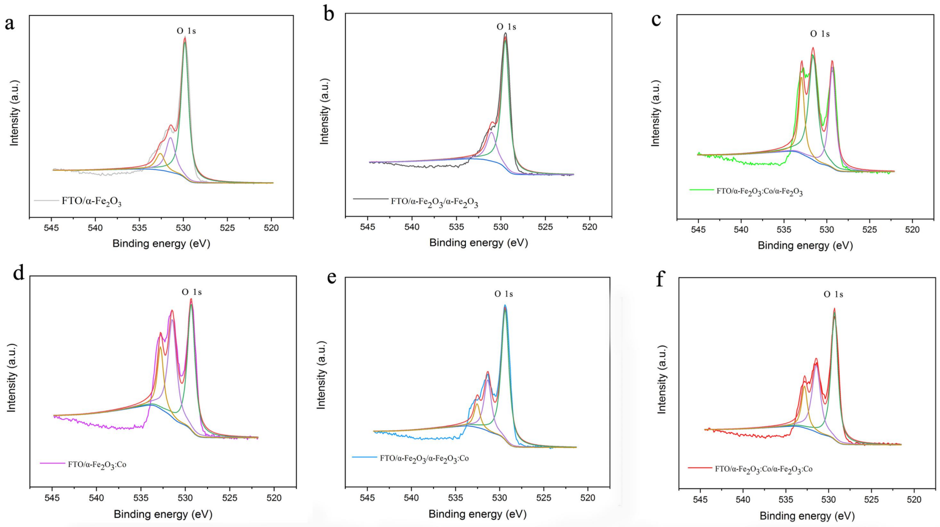

Fig. S3 O 1s XPS of (a) FTO/α-Fe_2O_3, (b) FTO/α-Fe_2O_3/α-Fe2O3, (c) FTO/α-Fe_2O_3:Co/α-Fe_2O_3, (d) FTO/α-Fe_2O_3:Co, (e) FTO/α-Fe_2O_3/α-Fe_2O_3:Co, and (f) FTO/α-Fe_2O_3:Co/α-Fe_2O_3:Co

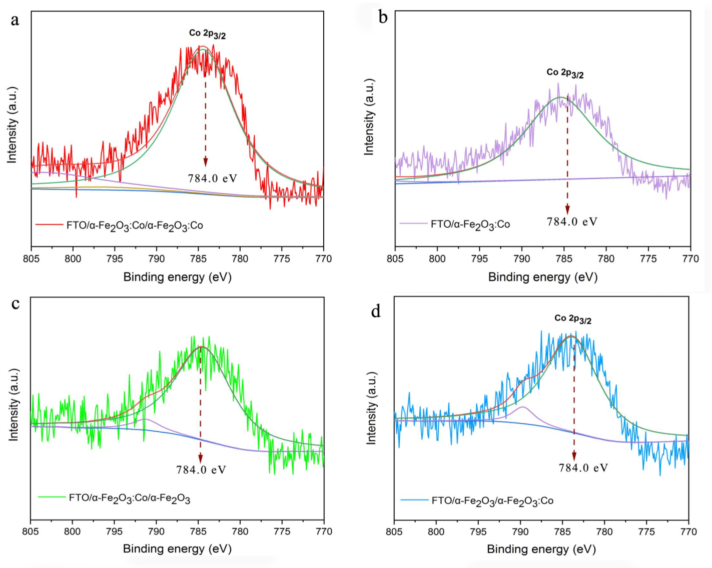

Fig. S4 Co 2p XPS of (a) FTO/α-Fe_2O_3:Co/α-Fe_2O_3:Co, (b) FTO/α-Fe_2O_3:Co, (c) FTO/FTO/ α-Fe_2O_3:Co/α-Fe_2O_3, and (d) FTO/α-Fe_2O_3/α-Fe_2O_3:Co

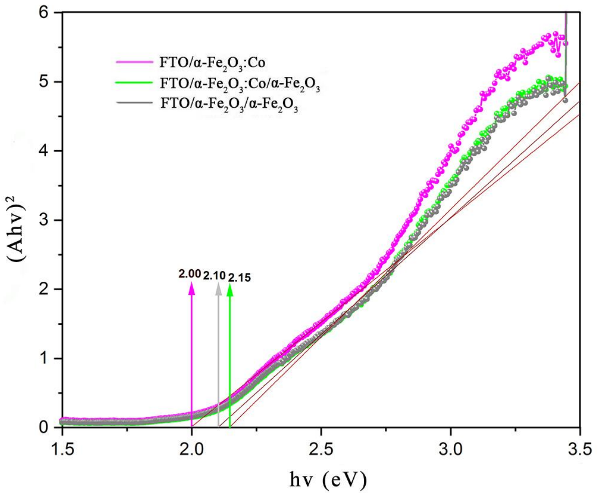

Figure S5 The DRS of (a) FTO/α-Fe_2O_3:Co/α-Fe_2O_3:Co, (b) FTO/α-Fe_2O_3:Co/α-Fe_2O_3, and (c) FTO/ α-Fe_2O_3/α-Fe_2O_3

Table S1 Photocurrent density of several electrodes at +1.0 V vs. SCE

Samples	F	FC	FF	FFC	FCF	FCFC
photocurrentdensity (mA/cm^2)	0.24	0.59	0.06	0 .998	1.37	0.96
ABPE (%)	0.19	0.43	0.09	0.93	1.25	0.86

Table S2 Current density of metal element doped Fe_2O_3 photoanodes

Samples	Photocurrent (mA/cm^2)	Potential (V vs. RHE)	Reference	Publication date
Undoped	0.83	1.23	Haiqing Ma et al.[1]	May, 2021
Zr-doped	1.23	1.23		
Undoped	0.40	1.23	Changhai Liu et al.[2]	May., 2019
Co-Pi-doped	1.11	1.23		
P-doped	0.67	1.23		
Undoped	0.61	1.27	Cheng Feng et al.[3]	Apr., 2020
Ni-doped	0.79	1.27		
Undoped	0.08	0.94	Dapeng Cao et al.[4]	Nov., 2020
Ti-doped	0.26	0.94		
Pristine hematite	0.033	1.23	Nyarige, Justine Sageka et al.[5]	Jan., 2022
Ag-doped	0.270	1.23		
Zn-doped	0.160	1.23		
Undoped	0.31	1.23	Wang Jun et al.[6]	Dec., 2017
Co-doped	0.55	1.23		
Sn-doped	0.94	1.23		
Undoped	0.018	1.23	Jiajia Cai et al.[7]	Feb., 2019
Ca-doped	0.095	1.23		
Undoped	0.60	1.7	Thimsen, E et al.[8]	Jan., 2011
Au-doped	1.12	1.7		
Undoped	0.24	1.47	—	Present work
Co-doped	0.59	1.47		
Co-doped (Double layer)	1.37	1.47		

E(RHE)=E(SCE)+0.0591pH+0.24;

Table S3 Equivalent circuit parameters of the different double layered photoanods

Samples	Rs ohm/cm^2	CPEbulkS sec^n/cm^2	Rtrap ohm/cm^2	CPEtrap S sec^n/cm^2	Rct ohm/cm^2
FF	17.53	8.69×10^{-5}	16983	1.09×10^{-4}	4379
FCF	13.05	1.49×10^{-4}	500.7	6.77×10^{-3}	2063
FFC	13.90	1.62×10^{-4}	844.4	1.63×10^{-3}	3819
FCFC	13. 13	1.45×10^{-4}	827.4	1.08×10^{-3}	1902

Rs is the seriesresistance; CPEbulk is the space charge capacitance; Rtrap is the bulk charge trapping resistance; CPEtrap is the surface trapstates capacitance; Rct is the charge transfer resistance across the semiconductor/ electrolyte.

Table S4 The calculated N_d of the different double layered photoanodes

Samples	FF	FFC	FCF	FCFC
Nd (cm^{-3})	3.35×10^{20}	3.22×10^{20}	2.51×10^{21}	8.62×10^{20}

Table S5 The comparisons of photoelectrochemical (PEC), electrochemical (EC), and photocatalytic (PC) processes on the reduction of Cr(VI)

	0 (min)	30 (min)	60 (min)	90 (min)	120 (min)	180 (min)
PC (%)	0	0.91	2.29	4.12	5.04	13.29
EC (%)	0	4.94	6.00	6.70	7.41	17.30
PEC (%)	0	10.03	59.76	67.06	73.44	84.85

Reference

[1] H.Q. Ma, J.B. Hwang, W.S. Chae, H.S. Chung, S.H. Choi, M.A. Mahadik, H.H. Lee, J.S. Jang (2021)Magnetron sputtering strategy for Zr-Fe_2O_3 nanorod photoanode fabricated from ZrOx/beta-FeOOH nanorods for photoelectrochemical water splitting, APPLIEDSURFACESCIENCE, 549. https://doi.org/10.1016/j.apsusc.2021.149233.

[2] C.H. Liu, Y. Xu, H. Luo, W.C. Wang, Q. Liang, Z.D. Chen (2019) Synthesis and photoelectrochemical properties of CoOOH/ phosphorus- doped hematite photoanodes for solar water oxidation, CHEMICAL ENGINEERINGJOURNAL, 363:23-32. https://doi.org/10.1016/j.cej.2019.01.112.

[3] F. Cheng, X.W. Li (2020) Effects of In Situ Co or Ni Doping on the Photoelectrochemical Performance of Hematite Nanorod Arrays, APPLIED SCIENCES-BASEL, 10. https://doi.org/10.3390/app10103567.

[4] D.P. Cao, J.B. Zhang, A.C. Wang, X.H. Yu, B.X. Mi (2020) Fabrication of Cr-doped $SrTiO_3$/Ti-doped alpha-Fe_2O_3 photoanodes with enhanced photoelectrochemical properties, JOURNAL OF MATERIALS SCIENCE & TECHNOLOGY,56:189-195. https://doi.org/10.1016/j.jmst.2020.04.025.

[5] J.S. Nyarige, A.T. Paradzah, T.P.J. Kruger, M. Diale (2022) Mono-Doped and Co-Doped Nanostructured Hematite for Improved Photoelectrochemical Water Splitting, Nanomaterials (Basel, Switzerland), 12. https://doi.org/10.3390/nano12030366.

[6] J. Wang, C. Du, Q. Peng, J. Yang, Y.W. Wen, B. Shan, R. Chen (2017) Enhanced photoelectrochemical water splitting performance of hematite nanorods by Co and Sn co-doping, INTERNATIONAL JOURNAL OF HYDROG ENENERGY, 42:29140-29149. https://doi.org/10.1016/j.ijhydene.2017.10.080.

[7] J.J. Cai, S. Li, G.W. Qin (2019) Interface engineering of Co_3O_4 loaded $CaFe_2O_4/Fe_2O_3$ heterojunction for photoelectrochemical water oxidation, APPLIED SURFACE SCIENCE, 466:92-98.https://doi.org/10.1016/j.apsusc.2018.10.022.

[8] E. Thimsen, F. LeFormal, M. Gratzel, S.C. Warren (2011) Influence of Plasmonic Au Nanoparticles on the Photoactivity of Fe_2O_3 Electrodes for Water Splitting, NANOLETTERS, 11:35-43.https://doi.org/10.1021/nl1022354.
